# Supplementary material for: Sickle Cell Disease: Metabolomic Profiles of Vaso-Occlusive Crisis in Plasma and Erythrocytes
Source: J Clin Med. 2020 Apr 11;9(4):1092. doi: 10.3390/jcm9041092 (PMC7230294; doi:10.3390/jcm9041092)
Supplement: Supplementary file 1 [file jcm-09-01092-s001.zip › jcm-768998-supplementary material 2/jcm-768998-for conversion -supplementary 2.pdf]

Supplementary Materials: Metabolomic Profiles of Vaso-Occlusive Crisis in Plasma and Erythrocytes

Klétigui Casimir Dembélé, Charlotte Veyrat-Durebex, Guindo Aldiouma, Stéphanie Chupin, Lydie Tessier, Yaya Goïta, Mohamed Ag Baraïka, Moussa Diallo, Boubacari Ali Touré, Chadi Homedan, Delphine Mirebeau-Prunier, Gilles Simard, Dapa Diallo, Bakary Mamadou Cissé, Pascal Reynier * and Juan Manuel Chao de la Barca

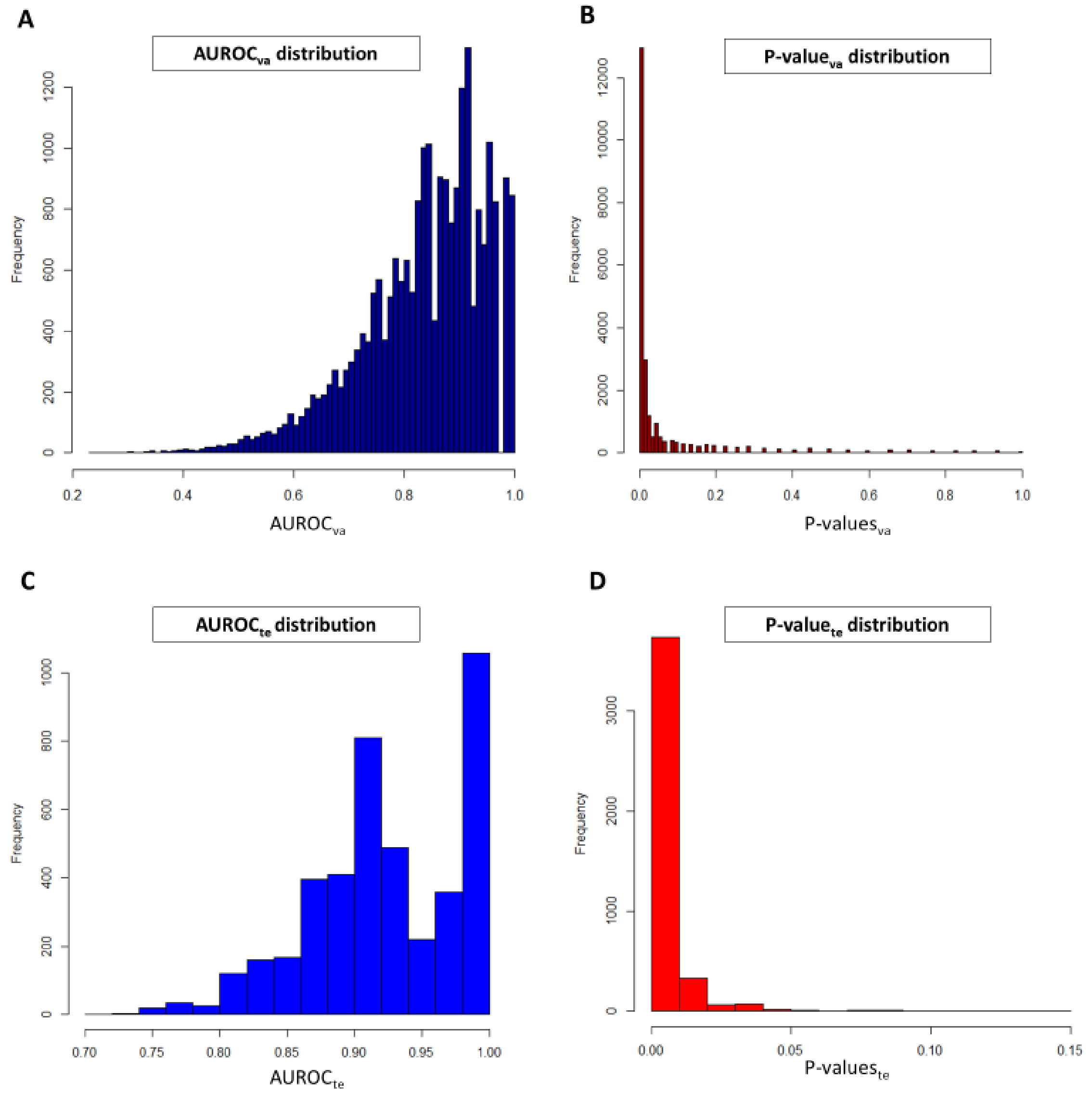

Figure S1. Distribution of AUROC (blue bars) and corresponding *p*-values (red bars) for PLSDA models obtained from plasma samples. Distributions are left-skewed for AUROCs and right-skewed for *p*-values. **A,** AUROC values of 23,345 models built with 60 samples of training-validation set. In each model, 40 samples (training set) have been used to build a PLSDA model and its predictive capability was tested with the 20 samples left (validation set). $AUROC_{va}$ represents the area under the ROC curve obtained for each model when tested in the validation set and *p*-$value_{va}$ in **B** indicates the probability for the correspondent model to be a random model not different from classifying each sample using coin tossing. Here left- and right-skewed distribution for AUROC and *p*-values, respectively indicate that PLSDA models predict significantly better that the random model does. **C, D**, Using 4277 best models (i.e., AUROCva ≥ 0.95) to predict class membership of 18 samples left in

the test set and using AUROC ($AUROC_{te}$) (**C**) and corresponding *p*-values (*p*-$values_{te}$) (**D**) as parameters for evaluating model predictive capability even better results compared to all PLSDA models built with the training-validation set are obtained. These excellent results in the test set increase confidence on predictive capabilities of BM.

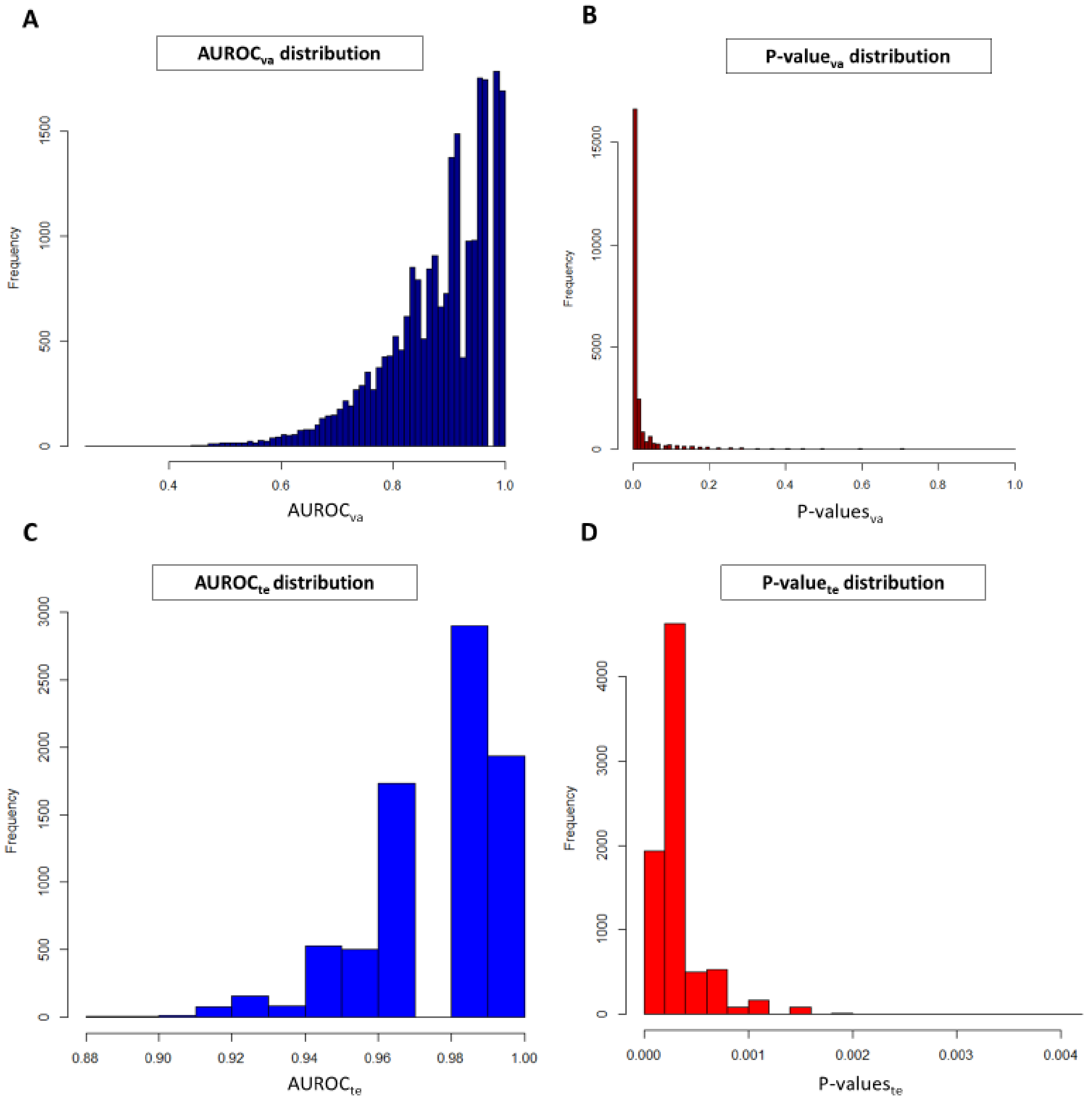

Figure S2. Distribution of the area under the ROC curve (AUROC)(blue bars) and corresponding *p*-value (red bars) for PLSDA models obtained from red cells samples. As for plasma samples distributions are left-skewed for AUROCs and right-skewed for *p*-values indicating that multivariate models predict significantly better than the random model. **A,** AUROC values of 23′345 models built with 60 samples of training-validation set. As for plasma samples each training model was built with 40 samples and its predictive capability was tested on the remaining 20 samples based on the area under the ROC curve ($AUROC_{va}$) and the associated *p*-$value_{va}$ in **B, C, D** For red cells best models sum up to 7′955 (i.e., AUROCva ≥ 0.95) and their performance in evaluating class membership of samples in the test set were excellent as measured by the AUROC ($AUROC_{te}$) (**C**) and the corresponding *p*-values (*p*-$values_{te}$) (**D**).
